# Complete mitogenome and phylogenetic analysis of the tropical rocky shore crab *Grapsus albolineatus* (Lamarck, 1818) (Crustacea: Grapsoidea)

**DOI:** 10.1080/23802359.2022.2136980

**Published:** 2022-11-04

**Authors:** Xiyue Wang, Shuyi Xu, Wenwen Chen, Xintong Hu, Lingxue Cui, Xue Li, Jiangyong Qu, Xumin Wang, Lijun Wang

**Affiliations:** College of Life Sciences, Yantai University, Yantai, China

**Keywords:** *Grapsus albolineatus*, mitogenome, phylogenetic tree analysis

## Abstract

In this study, the complete mitogenome of *Grapsus albolineatus* (Lamarck, 1818) (Crustacea: Grapsoidea) was sequenced. The mitogenome of *G. albolineatus* was a circular molecule with 15,578 bp length. Its nucleotide composition was 26.81% A, 16.37% G, 34.51% T, and 22.31% C. It comprised 13 protein-coding genes (PCGs), 22 transfer RNA (tRNA), and two ribosomal RNA (rRNA). All PCGs were initiated by ATN codons, except for the atp8 and nad1 genes. Ten PCGs used a common stop codon of TAA or TAG, and the other three ended with a truncated stop codon (a single stop nucleotide T). Phylogenetic analysis revealed that *G. albolineatus* was closely related to species from the genera *Pachygrapsus* and *Metopograpsus*.

The tropical rocky shore crab *Grapsus albolineatus* (Lamarck, 1818) (Crustacea, Decapoda, Grapsoidea), is a crucial species engineering coastal soil by its litter feeding activities. It is widely distributed along the rocky coastlines of China (Guangdong, Hainan, Taiwan), Japan, Hawaii, and Australia (Dai et al. 1986). The fluctuating asymmetry for leg segments of *G. albolineatus* can be applied as a developmental instability approach to identify stressed coastal area. In addition to maintaining ecosystem stability, the larvae of *G. albolineatus* are used as natural bait for aquaculture (Gao et al. 2000). The alcalase hydrolysis of rocky shore crab *G. albolineatus* can produce bioactive peptides with potent antioxidant and antibacterial activities as affected by the degree of hydrolysis up to a certain level (Shaibani et al. 2019). Peptide fractions isolated from the protein hydrolysate of *G. albolineatus* inhibited the proliferation of cancer cells and can be regarded as new agents for nutraceutical and pharmaceutical applications (Shaibani et al. 2020). The interest in the study of *G. albolineatus* has increased significantly; however, the research on this species is limited at the molecular level. Due to coastal land reclamation and commercial exploitation, the population genetic diversity of *G. albolineatus* is under threat. Therefore, it is necessary and urgent to perform genetic investigations on *G. albolineatus*. Thus, the complete mitogenome of *G. albolineatus* was sequenced in order to provide a theoretical basis for species identification and facilitate the protection of crab populations.

The samples used for sequencing were collected in Sanjiang Town, Haikou City, Hainan Province, China (N18°09′34″, E108°56′30″). The samples were preserved in 100% alcohol when collected, and then stored at −80 °C freezer for long-term storage (Pu et al. 2017). The specimen was deposited in the marine specimen repository of Yantai University (YTU-SKY-201900370102, contact person: Jiangyong Qu; email: qjy@ytu.edu.cn). Genomic DNA was extracted using column animal mtDNAOUT (Baiaolaibo Science Technology Co., Ltd., Beijing, China, BTN120501) and quality control was subsequently carried out on the purified DNA samples. The mitogenome sequence was loaded to an Illumina NovaSeq 6000 sequencing platform (Illumina, San Diego, CA) and assembled using SPAdes v3.10.1. The complete mitogenome was annotated using MITOS webserver (Bernt et al. 2013), subsequently improved by Geneious Prime, and phylogenetic tree was constructed by MrBayes 3.2 program (Ronquist et al. 2012).

The complete mitogenome of *G. albolineatus* (GenBank accession number: MT755825) was 15,578 bp. The nucleotide composition of this mitogenome was A = 4175 bp (26.8%), T = 5375 bp (34.5%), C = 3473 bp (22.3%), and G = 2555 bp (16.4%). Previous studies have shown similar findings in Crustacea, the GC content was lower than the AT content (Tsang et al. 2015; Song et al. 2020). The mitogenome contained 13 protein-coding genes (PCGs), that included seven NADH dehydrogenases (*nad*1, *nad*2, *nad*3, *nad*4, *nad*4*L*, *nad*5, and *nad*6), three cytochrome c oxidases (*cox*1, *cox*2, and *cox*3), one cytochrome b (*cob*), and two ATP synthases (*atp*6 and *atp*8). Furthermore, it contained 22 transfer RNA (tRNA) and two ribosomal RNA (rRNA). The total length of the 13 PCGs was 11,163 bp. Seven of the 13 PCGs (*nad*4, *nad*4*L*, *nad*5, *cox*1, *cox*2, *cox*3, and *cob*) started with ATG, *nad*2 and *atp*6 started with ATT, *nad*3 started with ATC, and *nad*6 started with ATA; however, the initiation codon of *atp*8 and *nad*1 could not be determined. Eight of the 13 PCGs had the stop codon TAA (*nad1*, *nad*3, *nad*4*L*, *nad*5, *nad*6*, atp*6, *atp*8, and *cox*3), and two had TAG (*nad*2 and *nad*4). It should be noted that the other three PCGs (*cox*1, *cox*2, and *cob*) terminated with an incomplete stop codon, which was completed by the addition of 3′ A residues to the mRNA. ‘Truncated or incomplete stop codon’ also exists in *Leptestheria brevirostris*, *Gondwanalimnadia* sp., and *Upogebia major* (Tladi et al. 2020; Emami-Khoyi et al. 2021; Sun and He 2021). Two rRNA genes, *12SrRNA* (827 bp) and *16SrRNA* (1402 bp) are separated by a trnV-GTA. All tRNAs had a typical cloverleaf structure and their length ranged from 63 to 72 bp.

To clarify the phylogenetic position of *G. albolineatus*, the mitogenome sequences of 18 closely related species and one outgroup species *Euphausia superba* were downloaded from GenBank database. Thirteen shared PCGs in each of the 20 complete mitochondrial genomes were aligned to generate a phylogenetic tree based on the Bayesian inference (BI) method by Mr. Bayes. Support values for each node were calculated using Bayesian posterior probability (BPP). The results confirmed that *G. albolineatus* was clustered with *G. tenuicrustatus* and was rooted in other Grapsoidea species (Figure 1). The genus *Grapsus* was most closely related to the genera *Pachygrapsus* and *Metopograpsus*, both of which belong to the family Grapsidae, which have not been previously reported. Altogether, our results provided insights into the crustacean mitochondrial genome diversity and the evolution of decapods.

**Figure 1.**
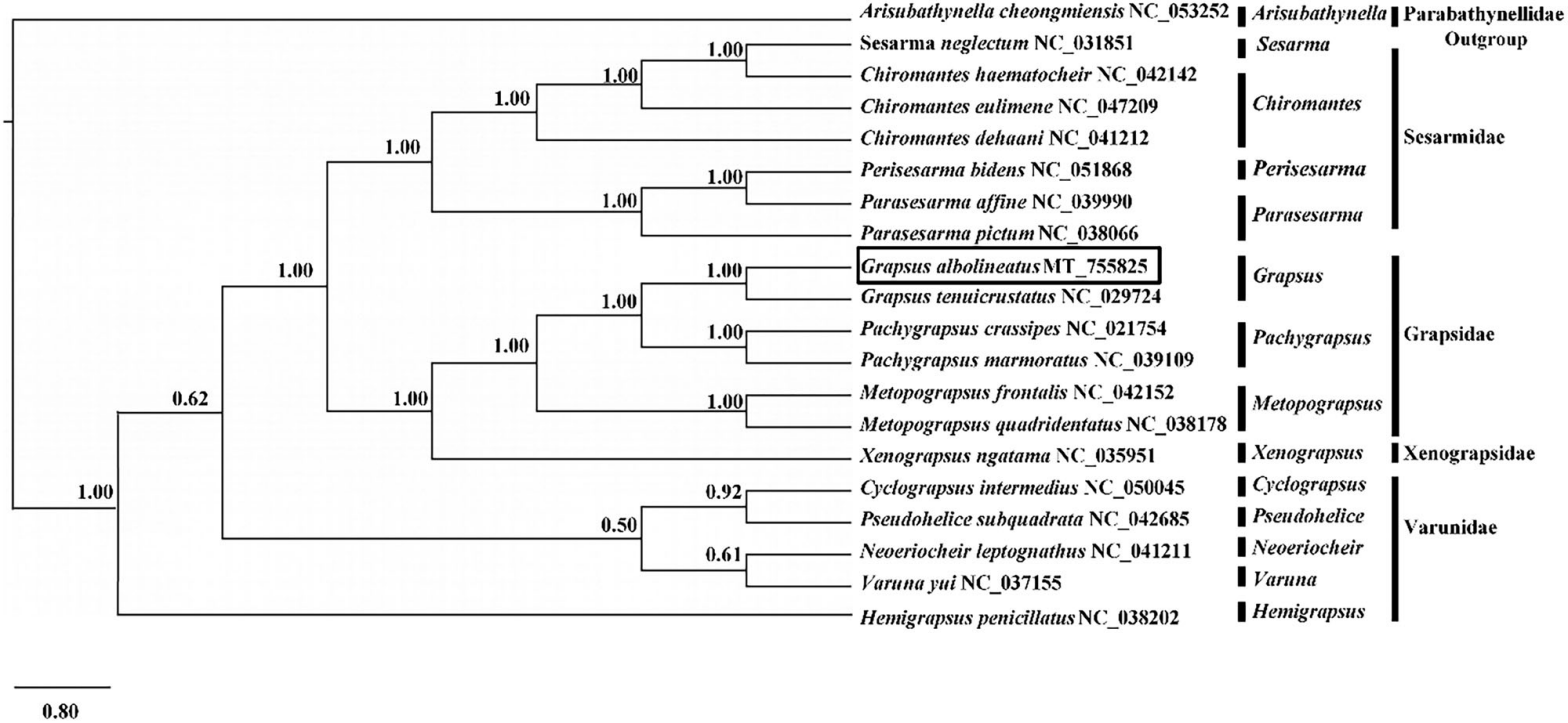
Phylogenetic position of *Grapsus albolineatus* based on mitogenome sequences. Nineteen in-group (Grapsoidea) and one out-group (Euphauiidae) were used for constructing this tree. The numbers after species’ names are the mitogenome GenBank accession numbers.

## Data Availability

All data in this study are openly available in GenBank (accession number MT755825). Original data submitted in NCBI, BioProject: PRJNA785768, SRA: SRR17110818, and BioSample: SAMN23576620.
